# Proteomic Comparison of *Entamoeba histolytica* and *Entamoeba dispar* and the Role of *E. histolytica* Alcohol Dehydrogenase 3 in Virulence

**DOI:** 10.1371/journal.pntd.0000415

**Published:** 2009-04-14

**Authors:** Paul H. Davis, Minghe Chen, Xiaochun Zhang, C. Graham Clark, R. Reid Townsend, Samuel L. Stanley

**Affiliations:** 1 Department of Medicine, Washington University School of Medicine, St. Louis, Missouri, United States of America; 2 Department of Molecular Microbiology, Washington University School of Medicine, St. Louis, Missouri, United States of America; 3 Department of Infectious and Tropical Diseases, London School of Hygiene and Tropical Medicine, London, United Kingdom; Jawaharlal Nehru University, India

## Abstract

The protozoan intestinal parasite *Entamoeba histolytica* infects millions of people worldwide and is capable of causing amebic dysentery and amebic liver abscess. The closely related species *Entamoeba dispar* colonizes many more individuals, but this organism does not induce disease. To identify molecular differences between these two organisms that may account for their differential ability to cause disease in humans, we used two-dimensional gel-based (DIGE) proteomic analysis to compare whole cell lysates of *E. histolytica* and *E. dispar*. We observed 141 spots expressed at a substantially (>5-fold) higher level in *E. histolytica* HM-1∶IMSS than *E. dispar* and 189 spots showing the opposite pattern. Strikingly, 3 of 4 proteins consistently identified as different at a greater than 5-fold level between *E. histolytica* HM-1∶IMSS and *E. dispar* were identical to proteins recently identified as differentially expressed between *E. histolytica* HM-1∶IMSS and the reduced virulence strain *E. histolytica* Rahman. One of these was *E. histolytica* alcohol dehydrogenase 3 (EhADH3). We found that *E. histolytica* possesses a higher level of NADP-dependent alcohol dehydrogenase activity than *E. dispar* and that some EhADH3 can be localized to the surface of *E. histolytica*. Episomal overexpression of EhADH3 in *E. histolytica* trophozoites resulted in only subtle phenotypic differences in *E. histolytica* virulence in animal models of amebic colitis and amebic liver abscess, making it difficult to directly link EhADH3 levels to virulence differences between *E. histolytica* and less-pathogenic *Entamoeba*.

## Author Summary

Infection with *Entamoeba histolytica* can result in disabling diarrhea or even death, while the morphologically identical and genetically similar *Entamoeba dispar* harmlessly colonizes the human intestine. Understanding the molecular differences between these two organisms by comparing their protein repertoire may help us to understand why *E. histolytica* invades into colonic tissue, while *E. dispar* remains a benign passenger. Here, we identify four proteins that appear to be differentially expressed between the two species and show that a metabolic enzyme, which would appear to be an unlikely candidate for a role in disease, is expressed at much higher levels in the pathogenic organism.

## Introduction

*Entamoeba histolytica*, a protozoan intestinal parasite, is the causative agent of amebic dysentery and amebic liver abscess [1], and is one of the leading causes of death from parasitic diseases. The closely related species, *Entamoeba dispar*, is morphologically indistinguishable from *E. histolytica* [2], and is highly prevalent in areas of poor sanitation. Importantly, *E. dispar* is a commensal and does not cause disease in humans, even in immunocompromised individuals. Previous studies have identified a number of *Entamoeba* molecules that appear to be linked to virulence, including cysteine proteinases, amoebapores, the Gal/GalNAc lectin and peroxiredoxin, but the virulence phenotype is unlikely to be secondary to only one, or even a few proteins [1], [3]–[8]. The ability to compare the genome and proteome of *E. histolytica*, and the related but nonpathogenic *E. dispar*, provides a powerful platform for more widespread screening for additional virulence factors of *E. histolytica*. Here we report the use of comparative proteomics of *E. histolytica* HM-1∶IMSS and *E. dispar* SAW760 to identify proteins that are differentially expressed between the two species, and the characterization of one of the differentially expressed proteins, EhADH3, identified by this screen.

## Materials and Methods

### *Entamoeba* species

*E. histolytica* HM-1∶IMSS and *E. dispar* SAW760 were grown axenically in LYI-S-2 with 15% adult bovine serum medium at London School of Hygiene and Tropical Medicine [9]. For proteomic analysis, approximately 5×10^6^ *E. histolytica* or *E. dispar* trophozoites were harvested and washed 3 times in ice-cold PBS to remove serum and medium proteins, then lysed in a buffer formulated to minimize post-lysis proteolysis (7 M Urea, 2 M thiourea, 4% CHAPS, 30 mM Tris, 5 mM magnesium acetate, 1× Roche Complete protease inhibitor cocktail with EDTA). Lysates were frozen at −80°C before analysis [3].

### 2-D difference gel electrophoresis (DIGE) and protein identification using tandem mass spectrometry

Trophozoite lysates were analyzed as previously described [3]. Briefly, lysates were thawed on wet ice and labeled with either Cy3 or Cy5 (GE Healthcare, Piscataway, NJ, USA) and quenched with lysine. The quenched Cy-labeled samples were then combined and added to an equal volume of 2× rehydration buffer (7 M urea, 2 M thiourea, 4% CHAPS, 4 mg/ml DTT) supplemented with 0.5% IPG (Immobilized pH gradient, GE Healthcare) buffer 3–11. Labeled protein extracts were separated by standard 2D gel electrophoresis. Following second-dimension focusing, the gel was fluorescently scanned using a Typhoon 9400 variable mode imager (GE Healthcare) to detect Cy3- and Cy5-specific emissions corresponding to protein concentration [10]. Fluorescent gel images were then analyzed using Decyder software (GE Healthcare), where individual spot volume ratios were calculated for each protein spot pair.

Gel features were selected in the DeCyder software, then excised and transferred to a 96-well source plate. The gel pieces were digested *in situ* with trypsin as previously described [11]. Spectra of the peptide pools were obtained on a MALDI-TOF/TOF instrument (ABI 4700) and operated as previously described [12] using peptides from trypsin autolysis (*m/z* = 842.51, 1045.56, and 2211.10) [13] for internal calibration. The most intense MS signals (n = 7–20) were automatically selected for tandem mass spectrometry using the MALDI-TOF/TOF instrument after exclusion of observed m/z values from contaminants. The peptide fragmentation spectra were processed (centroiding and background subtraction) with GPS Explorer and searched using MASCOT, V1.9 (Matrix Sciences, London) against the NCBI non-redundant database (26-07-2005 build date), which contains the published genome of *E. histolytica*.

Peptide pools from the gel features that were not identified using MALDI-MS/MS were analyzed using capillary reversed-phase HPLC-MS/MS using an electrospray-quadrupole time-of-flight mass spectrometer (Q-STAR XL, Applied Biosystems). Peptide pools from gel features that remained unidentified by either MALDI-MS/MS or quadrupole-TOF-LC-MS/MS were analyzed using nano-LC-linear-quadrupole ion trap Fourier transform ion cyclotron resonance mass spectrometry as previously described [14]. Identifying peptide information for the 4 proteins discussed in the text can be found in Table 1.

**Table 1:** Comparison of NADP-dependent alcohol dehydrogenase activity in lysates of *E. dispar*, *E. histolytica* HM-1∶IMSS, and *E. histolytica* HM-1∶IMSS overexpressing EhADH3 (HAO).

NADP-dependent ADH activity	*E. dispar*	HM-1∶IMSS	HAO
Units/mg	0.03±.02^1^	0.24±0.11^2^	0.85±0.31

Units/mg represents the enzyme activity (conversion of 1 mmole NADPH/min/mg of lysate protein using ethanol as substrate) within the lysates.

1 P≤0.004 for the difference in activity between lysates from *E. dispar* and *E. histolytica* HM-1∶IMSS.

2 P<0.001 for the difference in activity between lysates from *E. histolytica* HM-1∶IMSS and HAO.

### Expression and purification of recombinant EhADH3

Primers derived from the sequence of EhADH3 (Z48752.1) [15], forward -AAGGATCCATGACAATGCTTAATTTCACATA and reverse - TTCTCGAGTTAATAAATGCTATTAAGAATTTGGAGAT were used to amplify a EhADH3 transcript from HM-1∶IMSS genomic DNA. The fragment was inserted into pCR 2.1 TOPO vector (TOPO TA Cloning Kit from Invitrogen, Carlsbad, CA), cut by BamHI and XhoI and cloned into pGEX-6p-1. The plasmid was expressed under 0.05 mM IPTG induction in BL21- Codon Plus RIL from Stratagene (La Jolla, CA) at 18°C under shaking at 250 rpm for 48 h.

To purify recombinant EhADH3, 1 L of the transfected BL-21 *Escherichia coli* cells were harvested by centrifugation at 1,500×g for 30 minutes 4°C and resuspended in 35 ml PBS with a Protease Inhibitor Cocktail Tablet from Roche (Indianapolis, IN). The cell suspension was passed through a French press twice at 12,000 PSI, and then centrifuged at 20,000×g for 30 minutes at 4°C. The supernatants were frozen at −80°C for later use. For purification on the GST column, 500 ml supernatant was thawed overnight at 4°C, filtered with a 0.22 um filters from MilliPore (Temecula, CA), and loaded on a B-PER GST Fusion Protein Purification Kit from Pierce (Rockford, IL) according the manufacturer's instructions. Following column washings with 200 ml PBS, 10 ml Wash Buffer 1, 10 ml Wash Buffer2 and 10 ml PBS, 1.5 ml PBS containing 120 ul PreScission Protease from GE Healthcare was added to the column and incubated overnight at 4°C. The column was eluted with 1 ml PBS, and the total volume (2.5 ml) containing partially purified EhADH3 was dialyzed against 25 mM MES pH 6.0 (Buffer A) overnight at 4°C. This material was then loaded onto a Resource Q ion exchange column from GE Healthcare. After column washes with Buffer A, EhADH3 was eluted by a salt gradient created by the addition of increasing amounts (0% to 100%) of Buffer B (Buffer A with 1 M NaCl) to Buffer A run over the column over 30 minutes at flow rate 2 ml/min. Every 2 ml fraction was analyzed at UV 260 nm for protein concentration and those with the highest OD were pooled, dialyzed against 10 mM Tris HCl pH 7.3 and then concentrated by Amicon Ultra-4 Centrifugal Filter Units from MilliPore. Purity of the recombinant EhADH3 was assessed by SDS-PAGE.

### Determination of the ADH Activity in *E. histolytica* or *E. dispar* lysates, and analysis of the alcohol preference for recombinant EhADH3

*E. histolytica* or *E. dispar* lysates used for analysis of alcohol dehydrogenase activity were prepared as previously described [16]. Two hundred micrograms (total protein) of lysate or 10 ug of purified recombinant EhADH3 were added to a cuvette containing containing 50 mM Glycine/NaOH, pH 9.5, 50 mM MgSO_4_, 60 mM DTT, 0.2 mM NADP and 0.1 M of the substrate alcohol to a final volume of 1 ml. The rate of increase in absorbance was observed at 340 nm (1 OD = 6.22 mM cm^−1^ NADPH). A unit of enzyme activity was defined as one micromole of product formed per minute of incubation at room temperature.

### Transfection

In order to overexpress EhADH3 in strains *E. histolytica* HM-1∶IMSS and *E. histolytica* Rahman, plasmid pNeoCass was used to construct plasmid pNeoADH3 [17]. KpnI and BamHI sites on pNeoCass were restricted, and the following primers were used to amplify EhADH3 from *E. histolytica* HM-1∶IMSS cDNA: Forward- 5′ -AATTGGTACCATGACAATGCTTAATTTCACATATTAC - 3′, Reverse: 5′ - ATCCGGATCCTTAATAAATGTCATTAAGAATTTGGAG - 3′. The insert was then cut and ligated into pNeoCass yielding pNeoADH3. Transfection was performed as previously described [18]. Briefly, 1×10^6^ amebae were washed 2× with cold PBS, and once with cold, fresh cytomyx. The amebae were resuspended with 800 ul cytomyx and 60 ug DNA into a chilled 0.4 cm electroporation cuvette. A BioRad GenePulser XCell was set to 25 uF and 3000 V/cm. Two successive pulses were completed 30 s apart, and then amebae were placed in medium and grown for 72 h before G418 drug selection. These strains were maintained in culture medium BI-S-33 containing G418 as previously described [19].

### Live immunofluorescence staining

Approximately 2×10^5^ amebae were grown to log phase, chilled, pelleted at 4°C and 400×g for 1 min, and resuspended in 2 ml resuspension/blocking buffer, which consists of 50% v/v TYI-S-33 [9] minus antibiotics and bovine serum, 50% v/v PBS with 20% heat-inactivated pooled goat sera (Sigma-Aldrich, St. Louis, MO), and 0.02 mM E-64 protease inhibitor (Sigma-Aldrich), for 10 min at 4°C. Amebae were pelleted as before and resuspended in 400 ul resuspension buffer with 1∶100 polyclonal rabbit anti-EhADH3 (AnaSpec, San Jose, CA). For specificity studies, the 1∶100 anti-EhADH3 antibody was first incubated with 10 ug/mL purified recombinant EhADH3 for 1 h at 37°C prior to addition to the cell pellet. After 20 min incubation at 4°C, amebae were washed 3× as before with resuspension buffer. The pellet was then resuspended in 400 ul resuspension buffer with 1∶200 highly-adsorbed goat anti-rabbit IgG-AlexaFluor 488 conjugate (Invitrogen, Carlsbad, CA). Amebae were incubated for 20 min at 4°C, washed 3× with resuspension buffer, then once at 4°C with 1× PBS. The pellet was then resuspended in 4% paraformaldehyde in 1× PBS at 37°C for 1 h. Fixed, pelleted amebae were then resuspended in 1 drop of Slowfade Gold with DAPI (Invitrogen) and applied to a microscope slide. Microscopic images were captured under 63× oil-immersion magnification using a LSM510 Laser Scanning Confocal microscope (Carl Zeiss, Thornwood, NY). Antibody-blocked and unblocked images were captured using identical intensity settings.

### SCID mouse model of amebic liver abscess

All our research on mice was approved by the Washington University Animal Studies Committee (ASC), and was conducted under AAALAC and USDA guidelines. For studies of amebic liver abscess, 1×10^6^ *E. histolytica* HM-1∶IMSS or *E. histolytica* Rahman trophozoites overexpressing EhADH3 or an equivalent number of the parental control cells were directly inoculated into the liver of male, 6 to 8 weeks old BALB/c or SCID mice, as previously described [20]. After 24 h, animals were sacrificed, and the livers were removed and weighed. The abscessed region of the liver was cut out and weighed, and the percentage of liver abscessed was calculated.

### SCID-hu mouse model of colonic disease

Severe combined immunodeficient mice were engrafted in the subscapular region with human colonic xenografts as previously described [21]. Grafts were infected with an intraluminal inoculation of 1×10^6^ trophozoites (either the parental wild type strain, the pNeo control strain, or *E. histolytica* HM-1∶IMSS or *E. histolytica* Rahman over-expressing EhADH3) and infection was assessed 24 h later. To measure the integrity of the intestinal permeability barrier, 20 h after infection human intestinal xenografts were intraluminally inoculated with fluoresceinated dextran, and serum levels of fluoresceinated dextran were measured using a fluorescent plate reader 4 h later [22]. Grafts were removed at the time of sacrifice, and levels of MPO (as a marker for the influx of inflammatory cells into the graft) were measured according to our previously described assay [22]. For histologic analysis, sections of the human intestinal xenograft were fixed in formalin, sectioned and stained with haematoxylin and eosin as previously described [21].

## Results/Discussion

### DIGE proteomic comparison of whole cell lysates is reproducible and varies little between *E. histolytica* HM-1∶IMSS isolates

Two-dimensional difference gel electrophoresis (2D-DIGE) allows protein lysates to be fluorescently labeled in such a way as to allow visualization of multiple channels representing up to three biological samples in a single physical gel while maintaining the ability to analyze chosen protein spots using modern mass spectrometric techniques [23]. The power of DIGE technology is based on the elimination of false signals created when comparing biological samples across separate acrylamide gels. In the past, DIGE analysis has been useful in understanding changes to biological systems following the application of drug or other stimuli, or to compare cancerous and precancerous tissue [24]. However, only a few recent efforts have been directed at comparing different species or strains of organisms [25]. The difficulties involving cross-species analysis using two-dimensional gel electrophoresis are not insignificant. Genomic differences resulting in amino acid substitutions, splice variants, post-translational modifications, truncations, or insertions can affect whether a protein spot location accurately reflects the coordinates of both species' protein due to resultant differences in molecular weight and isoelectric point. In addition, if complete genomic information is not available for one or both of the species under comparison, it may be difficult to identify those cases where the primary amino acid structure of a given protein differs significantly between the species. These issues were felt to be factors in a recent attempt to use conventional 2-dimensional gels to delineate proteomic differences between *E. histolytica* and *E. dispar* [26]. The recent progress on the genome of both *E. histolytica* and *E. dispar* has significantly reduced, but not completely eliminated these issues for this comparison.

**Figure 1:**
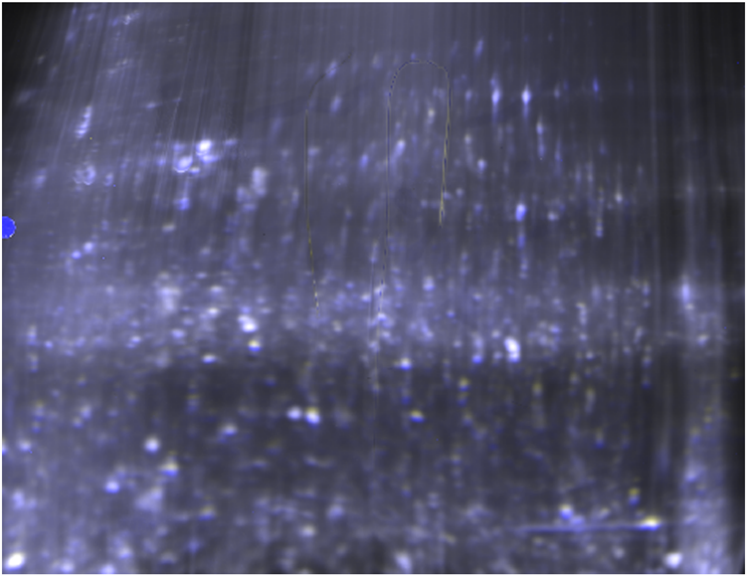
2D-DIGE comparison of two *E. histolytica* HM-1∶IMSS strains. 2852 spots were identified in this gel representing whole cell lysates from two strains of *E. histolytica* HM-1∶IMSS separately prepared. One was maintained in Saint Louis, Missouri, USA, and the other in London, England. Using a three-fold cutoff, only 6 labeled protein spots were found to fluoresce at different levels, suggesting limited biological variation exists between preparations and isolates. White spots are indicative of identical protein amounts; blue represents increased abundance in the Saint Louis isolate, while yellow represented increased abundance in the London isolate.

To test the reliability of the DIGE approach, we first performed comparative DIGE on identical samples of *E. histolytica* HM-1∶IMSS lysates, varying only the concentration of protease inhibitor added to the cell lysate (doubled in the second sample aliquot). No differences in spots were observed when a cutoff of 3-fold difference in fluorescent intensity was used, indicating that inter-sample full-length protein level differences secondary to endogenous proteases were not important at these protease inhibitor concentrations. To measure potential differences in spots based on sample preparation, lysates from the same *E. histolytica* HM-1∶IMSS isolate were captured one week apart, both in the logarithmic phase of growth. Again, with a 3-fold cutoff for differences, only 1 protein spot differed from more than 2800 computationally indexed spots (a five-fold cutoff reduces the number of observed differences to zero). Finally, we compared lysates from *E. histolytica* HM-1∶IMSS separately maintained in two distant laboratories (“Saint Louis” and “London”) using separate growth media (Figure 1). Out of nearly 2900 spots, only 6 differed using a 3-fold cutoff (1 differed at a 5-fold cutoff). These 6 spots were not identified by mass spectroscopy, but based on gel location they were not the same as those subsequently identified in this *E. histolytica* and *E. dispar* comparative study (Figure 1). These data indicate that the differences observed between species in the DIGE experiment are unlikely to be due to random proteolysis, differences in clonal populations, medium, or lysate preparation. This is especially true when a strict cut-off of 5-fold is used to identify differences between two samples derived from separate species.

### Proteomic comparison of *E. histolytica* HM-1∶IMSS and *E. dispar* SAW760 using DIGE

**Figure 2:**
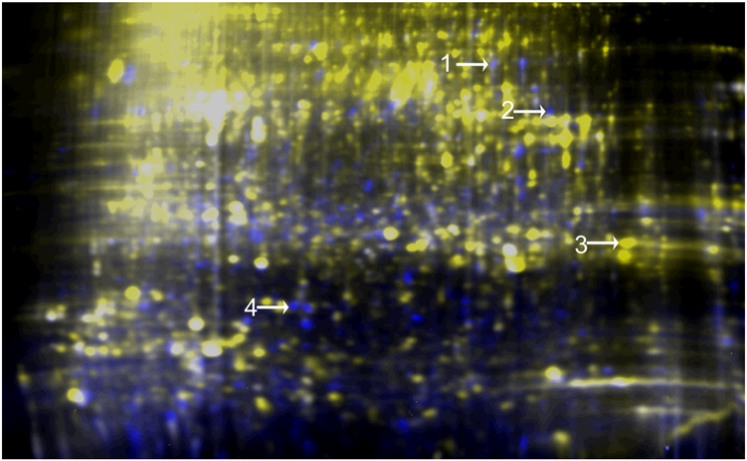
Representative 2D-DIGE gel of *E. histolytica* and *E. dispar* lysates demonstrating the extent of differences between species. One representative gel image of DIGE comparisons between *E. histolytica* and *E. dispar* highlights the measured differences in fluorescently labeled protein abundance between these two species. Yellow identifies protein spots that were proportionately higher in *E. dispar*; blue represents increased abundance in *E. histolytica*; white represents equal signal. Proteins were identified as follows (see Table 1): 1: ADH2- Higher in *E. histolytica* 6.1×; 2: ADH3- Higher in *E. histolytica* 5.8×; 3: Grainin 2- Higher in *E. dispar* 9.6×; 4: LIM domain protein- Higher in *E. histolytica* 12.6×.

We used 2D-DIGE to compare the proteomes of *E. histolytica* HM-1∶IMSS and *E. dispar* SAW760. We were able to resolve an average of 2676 spots (+/−109 spots) in three 2-D gels each containing distinct biological replicates of lysates from each species. The number of differentially expressed spots was a function of the cut-off used for differential expression, but requiring a minimum of 5-fold differential expression yielded an average of 141 (+/−16) spots expressed at a higher level in *E. histolytica* HM-1∶IMSS than *E. dispar*, with an average of 189 (+/−42) spots showing the opposite pattern. Figure 2 is an image of one representative gel. Selecting only a subset of these results for mass spectrometric analysis and using the strictest criteria (5-fold or greater difference in intensity, and spots that were reproducibly identified by mass spectrometry in at least two replicates) we found 4 proteins that could be unequivocally identified that showed differential expression between *E. histolytica* HM-1∶IMSS and *E. dispar* SAW760 (see Table 1 for peptide identification).

**Figure 3:**
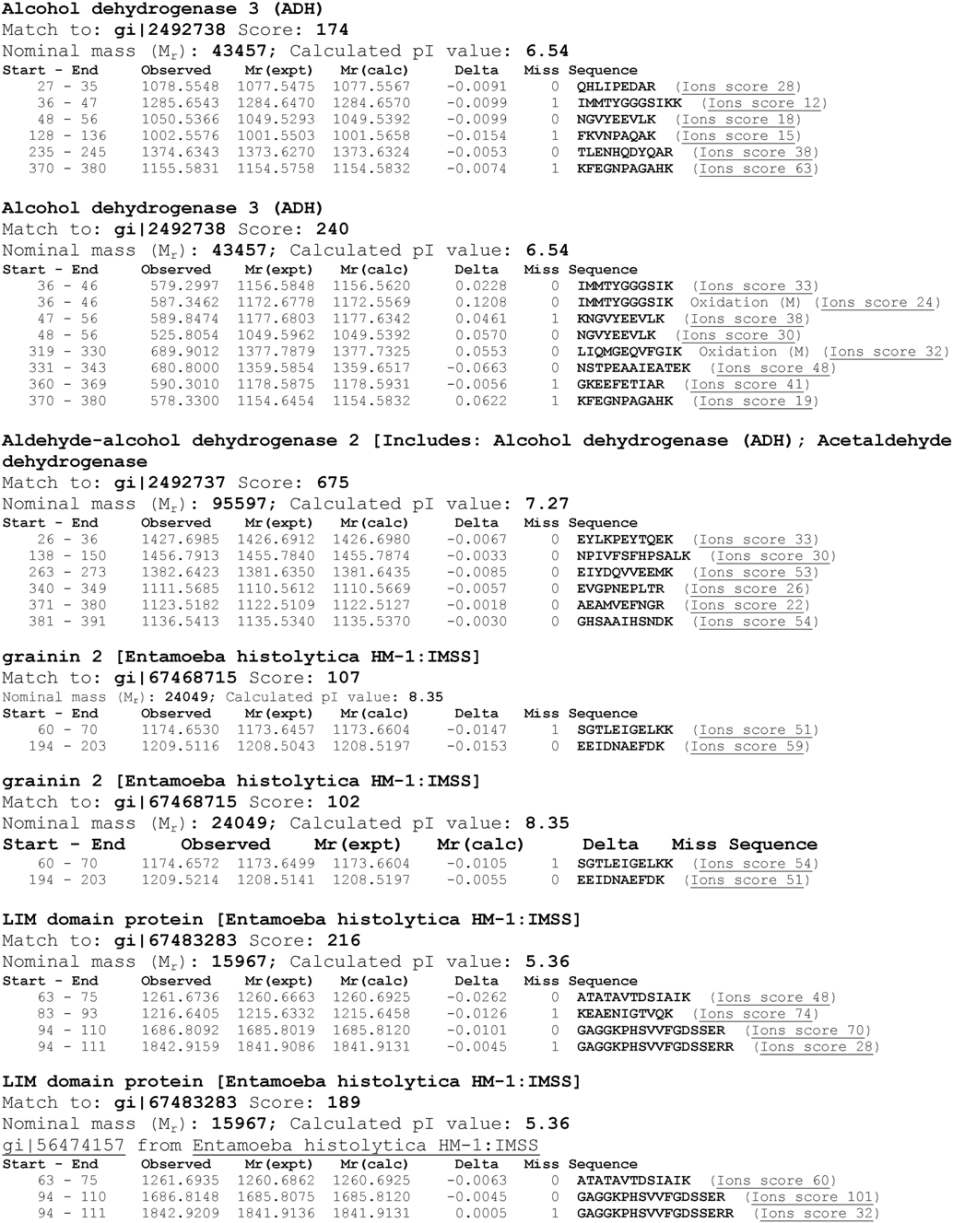
Peptide data from observed proteomic differences between *E. histolytica* and *E. dispar*.

Three proteins were present at higher levels in *E. histolytica* HM-1∶IMSS, and one was found at higher levels in *E. dispar* (Figure 2). Strikingly, three of these proteins were identical to ones identified as differentially expressed in the proteomic comparison between *E. histolytica* HM-1∶IMSS and the less virulent strain *E. histolytica* Rahman (Figure 3) [3]. The novel protein grainin 2 (gi67468715) was expressed at higher levels in *E. dispar* than in *E. histolytica* HM-1∶IMSS, with an average increase of 11.8-fold. Grainins are calcium binding proteins of unknown function found in *E. histolytica* granules [27]. A homology search of the provisional *E. dispar* genomic database [28] showed the inferred homologous *E. dispar* protein is 94% identical and 98% similar to the *E. histolytica* protein (EDI_060410). We postulate that increased grainin levels may contribute to a reduced virulence phenotype, since grainin levels were decreased in trophozoites recently obtained from amebic liver abscesses, and were higher in the proteome of the reduced virulence strain *E. histolytica* Rahman compared to *E. histolytica* HM-1∶IMSS [3],[29]. The finding that grainin 2 is expressed at significantly higher levels in *E. dispar* compared to *E. histolytica* is consistent with this hypothesis.

One of the proteins expressed at higher levels (average of 14.1-fold) in *E. histolytica* compared to *E. dispar* was a protein containing a LIM domain (gi67483283) (Figure 3). The LIM domain is a cysteine and histidine rich domain composed of two zinc fingers. LIM domains mediate protein-DNA and protein-protein interactions and function in tissue differentiation, cytoskeletal rearrangements, and other regulation of transcription [30]. A search of the provisional *E. dispar* sequence database yielded a truncated gene (77% complete) which is 99% identical to the *E. histolytica* gene (EDI_092410). In addition, the full length *E. histolytica* gene is nearly identical to the LIM domain protein found elevated in *E. histolytica* strain HM-1∶IMSS when compared to strain *E. histolytica* Rahman [3].

The two other proteins found at higher levels in *E. histolytica* HM-1∶IMSS than *E. dispar* were alcohol dehydrogenase 2 and alcohol dehydrogenase 3 (gi2492737 and gi2492738, respectively) (Figure 3). This finding was of interest, given a prior report that a virulent strain of *E. histolytica* showed higher alcohol dehydrogenase activity than a less virulent strain [15]. *E. histolytica* alcohol dehydrogenase 2 (EhADH2) [31],[32] is a NADH and iron-dependent bifunctional alcohol dehydrogenase and acetaldehyde dehydrogenase whose activity is absolutely required for *E. histolytica* fermentation, growth and survival [16]. EhADH2 was originally isolated as a laminin-binding protein from *E. histolytica* lysates, but there is no direct experimental evidence that it serves this function *in vivo* [31].

### Characterization of *E. histolytica* ADH3

**Figure 4:**
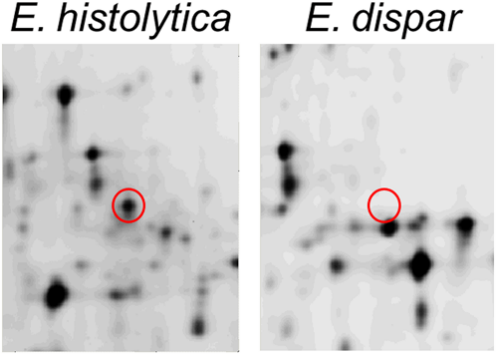
The gel area and spot representing ADH3 from one representative DIGE gel. The right panel is a fluorescent intensity scan of *E. histolytica*; the identical region from *E. dispar* is on the left. The outlined spot was identified as ADH3 by mass spectrometry. The demarcated region was used to calculate the signal fold difference between the species' ADH3 protein abundance, which was 5.82-fold higher in *E. histolytica* than *E. dispar* in this gel.

**Figure 5:**
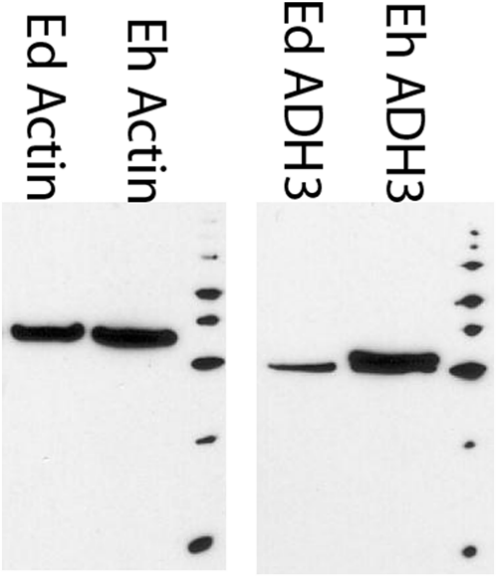
Anti-ADH3 Western blot confirms the difference between ADH3 protein abundance between species. Polyclonal antibodies developed against recombinant EhADH3 were generated in rabbits, and used to stain amebic lysates on a 1D Western blot. Densitometric analysis, normalized against the amount of actin present in each species' sample, results in 5.4-fold more ADH3 in *E. histolytica* compared to *E. dispar*. To the right of each image is MagicMark XP (Invitrogen, Carlsbad, CA) marking the following ascending molecular weights: 20 kD, 30 kD, 40 kD, 50 kD, 60 kD, 80 kD, 100 kD, and 120 kD.

*E. histolytica* alcohol dehydrogenase 3 is an NADPH- dependent alcohol dehydrogenase whose physiologic role in *E. histolytica* metabolism remains unknown [33]. The most recent *E. histolytica* resequencing data show that EhADH3 has no closely related inparalog (the closest, EHI_088020 is only 70% identical), differing from initial reports suggesting multiple copies of this gene. Our DIGE analysis (Figure 4) indicated an average increase of 8.6-fold in EhADH3 levels *in E. histolytica* compared to two nearly identical *E. dispar* homologs (EDI_307670 and EDI_09820) which are 90% identical and 95% similar to *EhADH3* in derived amino acid sequence. This difference in expression was confirmed by immunoblotting using polyclonal sera that showed EhADH3 was present at 5.4-fold higher levels in *E. histolytica* compared to *E. dispar* by densitometry (Figure 5). Additionally, we recently found that EhADH3 is present at significantly higher levels in *E. histolytica* HM-1∶IMSS than the reduced-virulence *E. histolytica* Rahman, and here there was no amino acid sequence difference between the predominant EhADH3 allele in *E. histolytica* HM-1∶IMSS and *E. histolytica* Rahman [3]. Taken as a whole, these data are consistent with *E. histolytica* EhADH3 (gi2492738), being present at higher levels in *E. histolytica* than its close homologues in *E. dispar*, but we cannot exclude the possibility than other more distant alleles of EhADH3 and EdADH3 could show different expression patterns.

**Figure 6:**
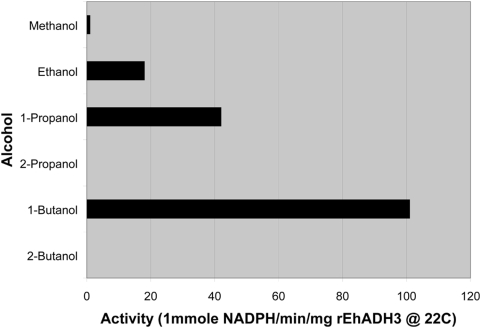
Recombinant EhADH3 prefers straight chain alcohols as a substrate. An enzymatic substrate preference assay was conducted to determine the optimal substrate for recombinant EhADH3. Butanol was demonstrated to be the preferred substrate, followed by shorter straight-chain alcohols. Branched alcohols were not detectably processed by recombinant EhADH3.

To determine whether there is physiologic evidence that ADH3 levels are higher in *E. histolytica* than *E. dispar*, we first analyzed purified recombinant EhADH3 to determine the optimal substrate specificity for the ADH3 enzyme. As shown in Figure 6, we found that EhADH3 prefers short chain unbranched alcohols as substrates, with the most activity using butanol. These data differentiate it from the NADP-dependent EhADH1 protein described by Kumar et al. [34], which preferred branched chain alcohols (2-propanol). We then measured the NADP-dependent ADH activity using ethanol as substrate in lysates from *E. histolytica* and compared it to that found in protein-matched lysates from *E. dispar*. As shown in Table 1, we found almost 8-fold more NADP-dependent ADH activity with ethanol as substrate in lysates of *E. histolytica* HM-1∶IMSS than in lysates of *E. dispar*. These data are consistent with higher levels of EhADH3 in *E. histolytica* than *E. dispar*, but we cannot be absolutely certain that all of the NADP-dependent ADH activity in these lysates (with ethanol as substrate) is secondary to the *Entamoeba* ADH3 enzyme family.

**Figure 7:**
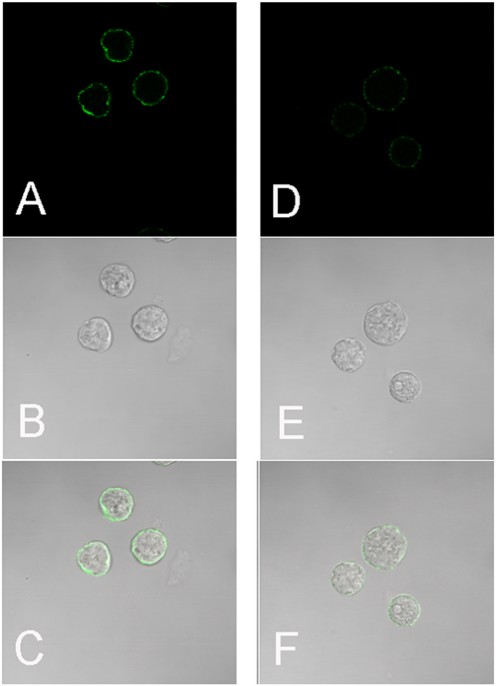
Live immuofluoresent surface staining of EhADH3 reveals its presence on the plasma membrane surface of *E. histolytica* HM-1∶IMSS. Amebae were harvested at 4°C, then blocked with blocking buffer for 10 min prior to staining with rabbit polyclonal anti-EhADH3 antibodies (panels A,B,C) or staining with antibodies pre-incubated with a molar excess of recombinant EhADH3 (panels D,E,F). Panels A and D show staining with the AlexaFlour secondary antibody, panels B and E the brightfield image, and panels C and F are a merge of the two. Magnification 63×.

As a first step towards determining whether EhADH3 could play a role in *E. histolytica* virulence we performed immunolocalization studies using polyclonal antiserum to recombinant EhADH3 with live trophozoites. As shown in Figure 7, some EhADH3 was localized to the surface of live *E. histolytica* HM-1∶IMSS trophozoites, and pre-incubation of the anti-EhADH3 antibodies with recombinant EhADH3 prior to staining significantly inhibited their ability to bind to the trophozoites' surface. These data indicate that at least some of the EhADH3 is on the surface of *E. histolytica* trophozoites, increasing the likelihood of interactions with host molecules. We used the same antibodies to determine whether ADH3 could be detected on the surface of *E. dispar*, and saw a similar staining pattern, but with much decreased overall intensity (data not shown).

We also explored whether EhADH3 might play some role in trophozoite adherence to host cells or host macromolecules due to its presence on the amebic plasma membrane. Some bacterial dehyrogenases have also been surface-localized and linked to adherence, including the *S. pneumoniae* 6-phosphogluconate-dehydrogenase, which mediates binding to buccal epithelial cells [35]. However, we were unable to show direct binding of EhADH3 to either sepharose-coupled fibronectin or laminin (data not shown), and polyclonal antiserum to recombinant EhADH3 failed to inhibit *E. histolytica* HM-1∶IMSS trophozoites from binding to Chinese Hamster Ovary cells (data not shown) [36].

**Figure 8:**
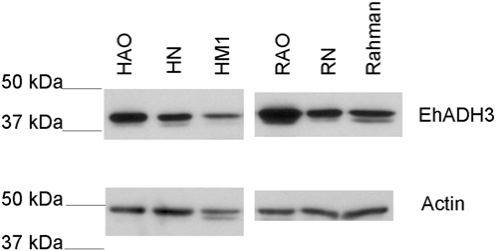
Western blot confirmation of EhADH3 overexpression in transfected *E. histolytica*. *E. histolytica* strain *E. histolytica* HM-1∶IMSS was transfected to overexpress EhADH3 Lysates from the parent strain (*E. histolytica* HM-1∶IMSS), the pNEO control transfectant (HN), and the transfectants overexpressing EhADH3 (HAO) were separated on an SDS-PAGE gel, blotted to PVDF, and stained with polyclonal anti-EhADH3 antibodies (top panel) or anti-actin antibodies (bottom panel). A similar experiment was performed for transfectants overexpressing EhADH3 in *E. histolytica* Rahman (right upper and lower panels). Lysates from the parent *E. histolytica* Rahman strain (Rahman), the pNEO control transfectant (RN) and tranfectants overexpressing EhADH3 were processed as above.

To further examine the potential role of EhADH3 in *E. histolytica* virulence, *E. histolytica* HM-1∶IMSS trophozoites and *E. histolytica* trophozoites of the reduced virulence *E. histolytica* Rahman strain were transfected with a plasmid designed to overexpress EhADH3. Immunoblotting of lysates from wild type and transfected trophozoites with antibodies to EhADH3 confirmed successful overexpression of EhADH3 in the transfectants (Figure 8). Based on densitometry, EhADH3 was overexpressed approximately 2-fold in transfected *E. histolytica* HM-1∶IMSS and *E. histolytica* Rahman. As a physiologic measure for EhADH3 overexpression, we quantified NADP-dependent alcohol dehydrogenase activity in lysates from wild-type or transfected *E. histolytica* HM-1∶IMSS trophozoites using butanol as a substrate. Transfected amebae had 3.1-fold more NADP-dependent alcohol dehydrogenase activity than the wild type control, confirming overexpression of functional enzyme (Table 1).

We then examined whether *E. histolytica* HM-1∶IMSS trophozoites overexpressing EhADH3 would show increased virulence in the SCID mouse model of amebic liver abscess. While we saw slightly larger abscesses in livers from mice inoculated with *E. histolytica* HM-1∶IMSS overexpressing EhADH3 (n = 11, mean abscess size of 38±16% of liver abscessed) compared with the parental wild type *E. histolytica* HM-1∶IMSS strain (n = 11, mean abscess size of 30±17% of liver abscessed), this difference was not statistically significant (P = 0.25). We then looked at whether overexpression of EhADH3 in *E. histolytica* Rahman would alter the ability of this strain to cause amebic liver abscesses in mice. We were unable to detect any increase in the size or presence of amebic liver abscesses in mice undergoing liver challenge with *E. histolytica* Rahman transfected with EhADH3 compared to challenge with either wildtype *E. histolytica* Rahman trophozoites or *E. histolytica* Rahman trophozoites expressing the pNEO control plasmid (data not shown).

In order to study phenotypic effects in colonic disease, we employed the SCID-hu human intestinal xenograft mouse model of amebic colitis [21]. We observed a statistically significant difference in the amount of intestinal inflammation (as measured by the levels of myeloperoxidase) in human intestinal xenografts infected with *E. histolytica* Rahman trophozoites expressing EhADH3 (N = 10, mean of 1.75±0.8 units MPO/mg total protein), compared to either human intestinal xenografts infected with either *E. histolytica* Rahman trophozoites transfected with the pNEO control plasmid (N = 10, mean of 0.8±0.7 units MPO/mg total protein, P<0.05) or wildtype *E. histolytica* Rahman trophozoites (N = 10, mean of 0.6±0.5 units MPO/mg total protein, P<0.01). Using our assay for damage to the intestinal permeability barrier [22], we were unable to detect any statistically significant differences between human intestinal xenografts infected with *E. histolytica* Rahman trophozoites expressing EhADH3 and human intestinal xenografts infected with either control, nor any obvious histological differences in sections from infected intestinal xenografts (data not shown). Interpretation of all of these results is complicated by the fact that we were only able to obtain an approximately 2-fold increase in EhADH3 production, and this may have been insufficient to detect a phenotypic difference between tranfectants and controls. Expression of antisense constructs did not reduce EhADH3 levels in *E. histolytica* HM-1∶IMSS (data not shown).

In summary, our proteomic comparisons identified a number of differences between the virulent *E. histolytica* HM-1∶IMSS and the commensal *E. dispar*. However, when we limited our analysis to those spots that showed the greatest magnitude difference and were consistently reproduced, we identified 4 proteins that were differentially expressed between the two species. Remarkably, 3 of the 4 were identical to proteins identified in a comparison of *E. histolytica* HM-1∶IMSS and the reduced virulence *E. histolytica* Rahman strain [3]. This may reflect both a potential role for these proteins in virulence, as well as their relative abundance and electrophoretic properties.

We focused our efforts on characterizing one of the proteins expressed at higher levels in *E. histolytica* HM-1∶IMSS, EhADH3. We found that EhADH3 does possess alcohol dehydrogenase activity but, unexpectedly, can be localized to the trophozoite surface. However, we were not able to detect an adherence function for EhADH3, and an approximate two-fold overexpression of EhADH3 in *E. histolytica* HM-1∶IMSS failed to result in increased virulence. A three-fold overexpression of the EhADH3 gene in the reduced virulence *E. histolytica* strain *E. histolytica* Rahman also did not lead to trophozoites capable of causing increased tissue damage in animal models of amebic liver abscess or amebic colitis; however, it did lead to an increased inflammatory response. Thus, while EhADH3 is more abundant in *E. histolytica* HM-1∶IMSS than either *E. dispar* or *E. histolytica* Rahman, we cannot directly link EhADH3 to the increased virulence of *E. histolytica* HM-1∶IMSS.
